# Migrant Women’s Help-Seeking Decisions and Use of Support Resources for Intimate Partner Violence in China

**DOI:** 10.1177/10778012211000133

**Published:** 2021-04-14

**Authors:** Ran Hu, Jia Xue, Xiying Wang

**Affiliations:** 1 University of Toronto, Ontario, Canada; 2 Beijing Normal University, China

**Keywords:** intimate partner violence, migrant women, help-seeking, China

## Abstract

In China, women who domestically relocate from rural or less developed regions to major cities are at a higher risk for intimate partner violence (IPV) than their non-migrant counterparts. Few studies have focused on Chinese domestic migrant women’s help-seeking for IPV and their use of different sources of support. The present study aimed to identify factors that influence migrant women’s help-seeking decisions. In addition, we also examined factors that contribute to migrant women’s use of diverse sources of support for IPV. A sample of 280 migrant women victimized by IPV in the past year at the time of the survey was drawn from a larger cross-sectional study conducted in four major urban cities in China, including Beijing, Shanghai, Guangzhou, and Shenzhen. Using a multinomial logistic regression model and a zero-inflated Poisson model, we found that factors influencing migrant women’s help-seeking decisions and their use of diverse sources of support included socioeconomic factors, IPV type, relationship-related factors, knowledge of China’s first anti-Domestic Violence Law, and perception of the effectiveness of current policies. We discuss implications for future research and interventions.

## Introduction

The mass domestic migration in contemporary China began in the early 1980s, a process characterized by a an ongoing relocation of people from rural or less urbanized regions to more developed urban areas. Currently, China has approximately 824 million urban residents (World Bank, 2018), of which about 244 million are domestic migrants who relocate and settle in economically vibrant cities from their hometowns (UNFPA, 2019). Although men constituted the vast majority of the migrant population in the early waves, over the last two decades, an increasing number of women have moved to urban cities for better education, economic opportunities, or reunification with their partners. The decrease in the sex ratio (male-to-female ratio, 15 and 64 years old) in the internal migrant population in China has reflected this trend: from 1.31:1 in 1990 to 1.18:1 in 2015 (UNFPA, 2019). With the backdrop of the ongoing domestic migration, in the field of IPV research in China attention has now been given to the prevalence of intimate partner violence (IPV) among migrant populations in urban areas (e.g., Chen et al., 2016; Tu & Lou, 2017). For instance, Tu and colleagues (2014) sampled 958 rural-to-urban migrant women in Shanghai and found that approximately 40% of the participants reported having experienced IPV in their lifetime. Another study that surveyed 1,744 married migrants in four cities in Zhejiang, an eastern coastal province in China, reported a rate of IPV of 44.2% in the past 12 months, and migrant women were more likely to experience sexual IPV compared to their male counterparts (Chen et al., 2016). Although these estimates are not representative of all urban-based migrant women, they suggest the seriousness of the issue of IPV among this population. Hence, more research should focus on this group of women.

The negative consequences of IPV on women are multifold. Studies conducted in different countries have reported increased risks for mental health symptoms, such as depression, posttraumatic stress disorders (PTSD), and suicidality (Ferrari et al., 2014; Gibbs et al., 2018; McLaughlin et al., 2012; Nathanson et al., 2012); different health concerns (Alhalal, 2018; Al-Modallal, 2016; Eaton et al., 2016; Massetti et al., 2017); and financial hardships and housing insecurity (Adams et al., 2013; Gilroy et al., 2016). Women face challenges and barriers when seeking help for IPV even though timely and sufficient support for IPV can significantly mitigate these risks (Grip et al., 2011; Guay et al., 2019; Rizo et al., 2017; Wong et al., 2016; Xue & Lin, 2020). Help-seeking for IPV is an ongoing and dynamic process (Kennedy et al., 2012; Liang et al., 2005). Liang et al. (2005) conceptualized help-seeking for IPV as a “dialectical process” (p.74), consisting of three interrelated stages: (a) recognizing a problem, (b) making a decision to seek help, and (c) selecting a helper (Liang et al., 2005). These “stages” should not be viewed as connected in a linear and strictly sequential fashion. Instead, they operate as a dynamic feedback loop that includes a series of interconnected thinking and action-taking. Informed by Andersen’s (1995) Behavioral Model of Health Service Utilization and the IPV help-seeking model by Liang et al. (2005), Kennedy et al. (2012) proposed a model of the help-attainment process to conceptualize formal help-seeking in women who have experienced sexual and physical victimization. In Kennedy et al.’s (2012) model, help attainment also contains multiple interrelated steps, such as appraising needs, seeking and accessing help, and assessing whether needs are met. These “process” models suggest that before a victim takes a help-seeking action, there is a crucial step in which the victim contemplates or thinks about whether a help-seeking action should be taken. According to the transtheoretical model (also known as the stages of change model, Prochaska & DiClemente, 1992), victims may neither take action nor think about seeking help at a pre-contemplation stage of IPV help-seeking. In contrast, at the stage of contemplation, victims would begin to recognize the violence “as a problem and consider pros and cons for taking action” (Liang et al., 2005, p. 74).

While the contemplation stage is a critical step in the process of help-seeking, it has seldom been examined in existing IPV studies of help-seeking decisions. For instance, a recent systematic review that assessed IPV help-seeking among women across varied cultural contexts showed that most studies used action-focused measures when examining help-seeking, such as victims’ reporting IPV to the police, utilizing medical support, and seeking social services (Satyen et al., 2019). Many studies have adopted a binary help-seeking decision as the outcome variable, such as seeking help versus not seeking help (e.g., Metheny & Stephenson, 2018) or formal help-seeking versus informal help-seeking (e.g., Ansara & Hindin, 2010; Cho et al., 2019). To fill the literature gap, the present study aimed to include in the analysis the stage of contemplation in IPV victims’ help-seeking process as an outcome variable when examining factors influencing victims’ help-seeking.

## Current Study

The aims of the present study were twofold. First, when examining influencing factors for victims’ IPV help-seeking decisions, we aimed to capture survivors’ internal contemplation on help-seeking in the analysis. Specifically, our first help-seeking outcome variable included three levels: (a) never thought about seeking help, (b) thought about seeking help but did not take actions, and (c) sought help for IPV. Second, the process models suggest that IPV help-seeking is by no means a one-time decision (Liang et al., 2005). Instead, the help-seeking feedback loop means a continuous thinking and decision-making process in which, as different needs arise, multiple different sources of support may be utilized either at different time points or simultaneously. Therefore, our second aim was to identify factors that contribute to migrant women’s use of diverse sources of support for IPV, measured by the total number of different forms of support sought after experiencing IPV.

While the help-seeking process manifests through a series of individual decisions, these actions are situated in a complex relational and social context. Therefore, a thorough examination of potential predictors should include sociodemographic characteristics, interpersonal or relational variables, and factors associated with the socio-cultural context of the population (Liang et al., 2005). A list of individual and relationship factors has been reported to be predictive of help-seeking behaviors, including age (Cho et al., 2017); income (Barrett et al., 2019; Barrett & St Pierre, 2011); education (Parvin et al., 2016; Tenkorang et al., 2016); employment status (Vives-Cases & La Parra, 2017); marital status (Linos et al., 2014); and different types of violence (Cho et al., 2017; Choi et al., 2018). In addition to including these important individual and relationship factors, in the present study we also included factors that reflect the geosocial context in which migrant women are situated, migration-related factors, and knowledge and beliefs related to the current anti-Domestic Violence (DV) Law in China.

## Method

### Procedures and Participants

The data for this study came from a larger study that aimed to examine workplace sexual harassment and IPV among migrant women in four major mega-cities in China, including Beijing, Shanghai, Guangzhou, and Shenzhen. We chose these four cities because they each are developed urban regions with large numbers of domestic migrants in China (UNFPA, 2019). Using a nonprobability sampling procedure, the research team recruited participants via Chinese social media platforms, including Weibo and WeChat, and from IPV- or DV-related listservs. The team developed a questionnaire, hosted by Wenjuanxing (www.wjx.cn), a Chinese survey website commonly used for data collection. Earlier studies have confirmed the usability of Wenjuanxing as a data collection platform for studies with similar populations (Hu et al., 2019; Lin et al., 2020). Data collection lasted about 2 months, from January 28 to March 31, 2018. The survey included three parts: (a) sociodemographic information, (b) migrant women’s experiences of sexual harassment at work, and (c) their experiences related to IPV.

Given that the present study focused on IPV, we did not analyze participants’ responses to questions related to sexual harassment. We used the following inclusion criteria to recruit participants: (a) 18 years of age or older, (b) self-identified as a woman, and (c) originally from a region outside of the four cities (i.e., Beijing, Shanghai, Guangzhou, and Shenzhen). Before proceeding to the survey questions, we provided participants with detailed information about the project and asked them to review it and provide electronic consent to participate in the study. Given the nature of the study, some participants, after disclosing their victimization, might hope to seek emotional and/or instrumental support from professional services. Therefore, the research team developed an electronic resource guide for participants who needed support upon completing the survey or at a later time. In the resource guide, we provided the introduction and contact information for legal, psychological counseling, and social service organizations that provide support to survivors of workplace sexual harassment and/or IPV, all of which are located in Beijing, Shanghai, Guangzhou, or Shenzhen. Using the same online survey platform, we distributed the resource guide to all participants. Upon completing the survey, participants each received RMB9.99 (approximately USD1.46) and were given an option to also participate in a lottery with a chance to win a RMB50 (USD7.1) gift card.

A total of 2,338 individuals responded and completed the survey, with 1,355 respondents meeting the inclusion criteria. Of the 1,355 respondents, 440 completed the sexual harassment section of the survey but were excluded from continuing to answer questions related to IPV, as they reported not being in an intimate relationship in the past year. A total of 750 out of 915 respondents completed the IPV section. They self-identified as heterosexual adult women with 280 having experienced IPV in the past 12 months at the time of the survey. Therefore, the 280 respondents comprised the final sample. Participants’ average age was 31.16 years (*SD* = 7.8, range = 18–65). While all participants reported having experienced at least one form of IPV in the past 12 months, psychological aggression (*n* = 143, 51.07%) was the most commonly reported form of IPV, followed by threatening and controlling behavior (*n* = 81, 28.93%), sexual violence (*n* = 66, 23.57%), and physical violence (*n* = 58, 20.71%). Over half of the participants migrated outside of their province (or town) of birth at the age of 18 to 30 years (*n* = 161, 57.5%). About half of the sample had lived in rural regions before migration (*n* = 146, 52.14%). Table 1 shows the sociodemographic characteristics of the sample and all covariates used in the analyses.

**Table 1.** Descriptive Statistics of the Sample (*N* = 280).

Variables	Total (*N* = 280) *n* (%)	Never thought about seeking help (*n* = 102) *n* (%)	Thought about it but did not seek help (*n* = 100) *n* (%)	Sought help (*n* = 78) *n* (%)
Age	*M* = 31.16 (*SD* = 7.8)	*M* = 32.29 (*SD* = 8.32)	*M* = 30.44 (*SD* = 7.17)	*M* = 30.59 (*SD* = 7.81)
Marital status				
In a dating relationship	90 (32.14)	33 (32.35)	30 (30.00)	27 (34.62)
Cohabiting	32 (11.43)	11 (10.78)	13 (13.00)	8 (10.26)
Married	127 (45.36)	52 (50.98)	41 (41.00)	34 (43.59)
Divorced or last relationship ended	31 (11.07)	6 (5.88)	16 (16.00)	9 (11.54)
Partner contact				
Almost saw each other everyday	141 (50.36)	59 (57.84)	43 (43.00)	39 (50)
On a weekly basis	77 (27.5)	24 (23.53)	31 (31.00)	22 (28.21)
On a monthly basis	29 (10.36)	5 (4.90)	15 (15.00)	9 (11.54)
Only saw each other on holidays	33 (11.79)	14 (13.73)	11 (11.00)	8 (10.26)
Broke up after IPV (*yes*)	123 (43.93)	33 (32.35)	59 (59.00)	31 (39.74)
Number of children				
0	150 (53.57)	48 (47.06)	58 (58.00)	44 (56.41)
1	75 (26.79)	29 (28.43)	21 (21.00)	25 (32.05)
2	47 (16.79)	21 (20.59)	18 (18.00)	8 (10.26)
3	7 (2.5)	3 (2.94)	3 (3.00)	1 (1.28)
4 or more	1 (0.36)	1 (0.98)	0 (0.00)	0 (0)
Educational level				
Completed middle school	22 (7.86)	11 (10.78)	9 (0.09)	2 (2.56)
Completed high school	46 (16.43)	16 (15.69)	14 (0.14)	16 (20.51)
Obtained an associate diploma	49 (17.50)	15 (14.71)	20 (0.20)	14 (17.95)
Completed college	114 (40.71)	36 (35.29)	41 (0.40)	37 (47.44)
Completed graduate school/above	49 (17.50)	24 (23.53)	9 (0.09)	9 (11.54)
Employment				
Full time	202 (72.14)	75 (73.53)	73 (0.73)	54 (69.23)
Part time	39 (13.93)	12 (11.76)	16 (0.16)	11 (14.1)
Unemployed	22 (7.86)	8 (7.84)	8 (0.08)	6 (7.69)
Other	17 (6.07)	7 (6.86)	3 (0.03)	7 (8.97)
Income				
2,000 Chinese Yuan or below	34 (12.14)	11 (0.11)	14 (0.14)	9 (11.54)
2,001–4,000 Chinese Yuan	55 (19.64)	25 (0.25)	18 (0.18)	12 (15.38)
4,001–6,000 Chinese Yuan	72 (25.71)	21 (0.21)	29 (0.29)	22 (28.21)
6,001–8,000 Chinese Yuan	52 (18.57)	17 (0.17)	20 (0.20)	15 (19.23)
8,001–10,000 Chinese Yuan	28 (10.00)	10 (0.10)	11 (0.11)	7 (8.97)
10,001 Chinese Yuan or above	39 (13.93)	18 (0.18)	8 (0.08)	13 (16.67)
Current living condition				
Self-owned home	58 (20.71)	29 (28.43)	13 (0.13)	16 (20.51)
Self-rented home	75 (26.79)	17 (16.67)	31 (0.31)	27 (34.62)
Co-rented with others	101 (36.07)	36 (35.29)	43 (0.42)	22 (28.21)
Stayed in home of others (e.g., friends)	16 (5.71)	6 (5.88)	5 (0.05)	5 (6.41)
Other	30 (10.71)	14 (13.73)	8 (0.08)	8 (10.26)
Residence status prior to migration				
Urban	134 (47.86)	47 (0.46)	48 (0.48)	39 (50)
Rural	146 (52.14)	55 (0.54)	52 (0.52)	39 (50)
Age first migrated for work (years)				
17 or below	17 (6.07)	6 (0.06)	4 (0.04)	7 (8.97)
18–30	161 (57.50)	58 (0.57)	67 (0.67)	36 (46.15)
31–40	32 (11.43)	15 (0.15)	4 (0.04)	13 (16.67)
41–50	59 (21.07)	18 (0.18)	22 (0.22)	19 (24.36)
51 or above	11 (3.93)	5 (0.05)	3 (0.03)	3 (3.85)
Number of cities ever migrated to				
1 to 2	130 (46.43)	50 (0.49)	39 (0.39)	41 (52.56)
3 to 4	125 (44.64)	40 (0.39)	55 (0.55)	30 (38.46)
5 or more	25 (8.93)	12 (0.12)	6 (0.06)	7 (8.97)
IPV victimization				
Psychological aggression (*yes*)	143 (51.07)	51 (50.00)	64 (64.00)	28 (35.90)
Controlling (*yes*)	81 (28.93)	30 (29.41)	34 (34.00)	17 (27.79)
Physical violence (*yes*)	58 (20.71)	23 (22.55)	26 (26.00)	9 (11.54)
Sexual violence (*yes*)	66 (23.57)	17 (16.67)	35 (35.00)	14 (17.95)
Belief that IPV is a private matter (*yes*)	80 (28.57)	32 (31.37)	31 (31.00)	17 (21.79)
Belongingness to current city (*yes*)	115 (41.07)	41 (40.2)	40 (40.00)	34 (43.59)
Belief that policies are effective (*yes*)	117 (41.79)	39 (38.24)	41 (41.00)	37 (47.44)
Knowledge of the anti-DV Law				
Never heard of it	38 (13.57)	21 (20.59)	10 (10.00)	7 (8.97)
Heard of it but don’t know much	144 (51.43)	54 (52.94)	54 (54.00)	36 (46.15)
Had some knowledge	83 (29.64)	26 (25.49)	30 (30.00)	27 (34.62)
Had comprehensive knowledge	15 (5.36)	1 (0.98)	6 (6.00)	8 (10.26)
Sources of support sought for IPV (*yes*)				
Friends/classmates	—	—	—	40 (51.28)
Family member(s)	—	—	—	45 (57.69)
Neighbor(s)	—	—	—	16 (20.51)
Colleague(s)	—	—	—	15 (19.23)
The police	—	—	—	13 (16.67)
Women’s Federation	—	—	—	20 (25.64)
Neighborhood committee	—	—	—	16 (20.51)
Mental health professional(s)	—	—	—	8 (10.26)
Lawyer(s)	—	—	—	20 (25.64)
Non-profit organization(s)	—	—	—	3 (3.85)
Mental health crisis hotline	—	—	—	10 (12.82)
Medical support	—	—	—	9 (11.54)
Social media/journalist(s)	—	—	—	7 (8.97)
Court(s)	—	—	—	5 (6.41)
Religious group(s)	—	—	—	4 (5.13)
Social worker(s)	—	—	—	5 (6.41)
Other(s)	—	—	—	5 (6.41)

*Note*. CNY = Chinese Yuan; ref. = used as the reference group; IPV = intimate partner violence.

### Measures

To identify factors that influence help-seeking decisions, we included five clusters of factors, all of which were treated as independent variables in our analyses: (a) experiences of IPV victimization, (b) sociodemographic information, (c) relationship factors, (d) migration-related factors, and (e) knowledge and beliefs regarding the current DV Law. Two help-seeking outcome variables were (a) help-seeking decision, a categorical variable, used in the multinomial regression model; and (b) the total number of different sources of support sought for IPV, a count variable, used in a zero-inflated Poisson (ZIP) regression model. These measures are detailed in the following sections.

#### IPV victimization

Four types of IPV victimization were included in our analyses: (a) psychological aggression, (b) threatening and controlling behavior, (c) physical violence, and (d) sexual violence. Participants were asked to report the occurrence of four types of IPV they endured in the past year at the time of the survey. To construct these measures, the research team consulted the Revised Conflict Tactics Scales (CTS2, Straus et al., 1996) and took into consideration the behavioral manifestations of IPV in the Chinese context and the ways these IPV-related behaviors are commonly framed in Chinese. A total of 15 items were developed to measure the four types of IPV. Since these items are different from the original CTS2, we conducted a factor analysis to identify the structure of the 15 items in a previously published study (Hu et al., 2019) in the context of China with a larger sample of heterosexual women (*N* = 1,301). Four factor dimensions (i.e., psychological aggression, threatening and controlling behavior, physical violence, and sexual violence) emerged through the analysis, and alpha values for the four factors ranged from .76 to .9, indicating excellent internal consistency.

Psychological aggression included three items: “neglecting,” “verbally humiliating or cursing,” and “talking ill or laughing at.” The threatening and controlling behavior included “verbally threatening,” “stalking or digital monitoring,” “threatening with self-harming or suicide attempts,” “restricting social interaction with family or friends,” “restricting physical freedom,” and “controlling financially.” Four items measured physical violence, including “slapping, pushing, or shoving;” “kicking, biting, punching, or choking;” “throwing sharp objects or using those to attack;” and “burning with boiling water or cigarettes.” Sexual violence was assessed by two items, “forcing to sexually touch or kiss” and “forcing to have sexual activities.” Three-point Likert-type scales were used for each question item (1 = *never*, 2 = *sometimes*, and 3 = *often*). In the present study, we dichotomized each one of the four IPV measures. Specifically, if the participant answered “never” to all the items on an IPV measure (e.g., the dimension of threatening and controlling), this IPV measure was coded 0, which represented that this type of IPV did not occur in the past year. When the participant answered “sometimes” or “often” to any of the items on an IPV measure, the measure was coded 1, which represented that such type of IPV occurred in the past year.

#### Sociodemographic information

Age was a continuous variable. Number of children was measured with an ordinal variable (0 = *no children*, 1 = *one child*, 2 = *two children*, 3 = *three children*, and 4 = *four or more*). Education was a categorical variable with five levels (1 = *completed middle school or below*, 2 = *completed high school*, 3 = *obtained an associate diploma*, 4 = *completed college*, and 5 = *completed graduate school or above*). Employment status was a categorical variable with four levels (1 = *full-time*, 2 = *part-time*, 3 = *unemployed*, and 4 = *other*). Income was measured with an ordinal variable (1 = *2,000 Chinese Yuan or below*, 2 = *between 2,001 and 4,000 Chinese Yuan*, 3 = *between 4,001 and 6,000 Chinese Yuan*, 4 = *between 6,001 and 8,000 Chinese Yuan*, 5 = *between 8,001 and 10,000 Chinese Yuan*, and 6 = *10,000 Chinese Yuan or above*). Current living situation was a categorical variable with five levels (1 = *self-owned home*, 2 = *self-rented home*, 3 = *co-rented with other[s]*, 4 = couch-surfing *at the home[s] of other[s]*, and 5 = *other*).

#### Relationship factors

Marital status included four categories (1 = *in a dating relationship*, 2 = *cohabiting*, 3 = *married*, and 4 = *divorced or last relationship ended*). Frequency of contact with the intimate partner included four levels (1 = *seeing each other every day*, 2 = *on a weekly basis*, 3 = *on a monthly basis*, and 4 = *only on holidays or vacations*). In addition, participants were also asked whether they broke up with the partner due to IPV (0 = *no* and 1 = *yes*) and whether they believed IPV is a private matter (0 = *no* and 1 = *yes*).

#### Migration-related factors

Age first migrated included five levels (1 = *17 or below*, 2 = *18–30*, 3 = *31–40*, 4 = *41–50*, and 5 *= 51 or above*). Number of cities ever migrated to included three levels (1 = *one to two cities*, 2 = *three to four cities*, and 3 = *five or more cities*). Region of residence before migration included two levels (1 = *rural* and 2 = *urban*). Having a sense of belonging to the local city of residence was coded binary (0 = *no* and 1 = *yes*).

#### Knowledge and beliefs regarding the current anti-DV Law

Respondents were asked how much they knew about the current anti-DV Law (1 = *never heard of it*, 2 = *heard of it but don’t know much*, 3 = *have some knowledge*, and 4 = *have comprehensive knowledge*) and whether they believed the current policies are effective in protecting their rights (0 = *no* and 1 = *yes*).

#### Help-seeking outcomes

The first outcome variable examined was migrant women’s help-seeking decision in response to IPV, measured categorically (1 = *never thought about seeking help*, 2 = *thought about seeking help but did not take actions*, and 3 = *sought help for IPV*). The second outcome variable was a count variable measuring the total number of different sources of help that were sought by participants. Respondents were asked to check whether they have sought help for IPV from the following sources of support: (a) friends or classmates, (b) family members, (c) neighbors, (d) colleagues, (e) the police, (f) the Women’s Federation, (g) neighborhood committee, (h) mental health professionals, (i) lawyers, (j) non-profit organizations, (k) mental health crisis hotline, (l) medical support, (m) social media/journalists, (n) courts, (o) religious groups, (p) social workers, and (q) other(s). Each one of the items was binary (1 = *yes*, 0 = *no*). The count variable was the sum of the total number of “*yes*” responses.

### Data Analysis and Model Selection

**Figure 1.**
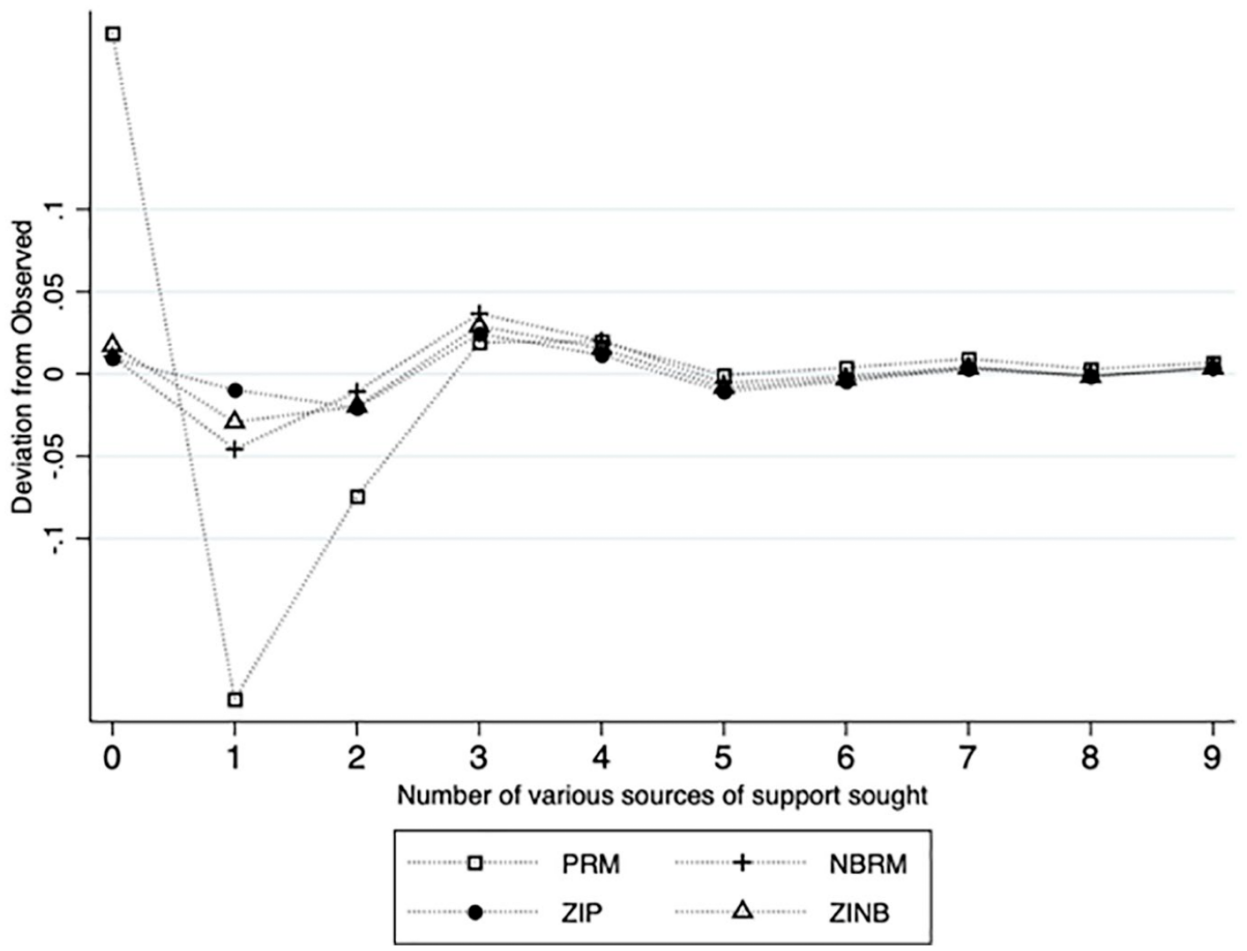
Residuals of the four count models. *Note.* PRM = poisson regression model; NBRM = negative binomial regression model; ZIP = zero-inflated Poisson model; ZINB = zero-inflated negative binomial model.

We first conducted a multinomial logistic regression analysis with the categorical help-seeking decision being the outcome variable (1 = *never thought about seeking help*, 2 = *thought about seeking help but did not take actions*, and 3 = *sought help for IPV*) to identify factors influencing the three decisions. We then used a ZIP regression model to identify factors associated with the total number of different forms of support that participants sought for IPV. We chose to use a ZIP model based on the following rationale. First, the dependent variable is a count variable with discrete positive numbers that represent the total number of different forms of help, a type of outcome variable for which either a Poisson regression model or a negative binomial model is more suitable, compared with Ordinary Least Squares regression (OLS; Coxe et al., 2009). Second, the count outcome variable, which in the present study is the total count of all different forms of support a woman sought for IPV, had excess zeros. For count-based outcome variables with a considerable number of zeros, a zero-inflated model, such as ZIP (one form of Poisson regression) or zero-inflated negative binomial (ZINB) model, may be suitable. Finally, we conducted four models, including a Poisson model (PRM), a Negative Binomial model (NBRM), a ZIP model, and a ZINB model, with the count outcome variable, and then used four types of model fit indices to select the model that best fit the data. Figure 1 plotted the residuals of each one of the four count models (PRM, NBRM, ZIP, and ZINB). A line closer to zero indicates smaller residuals and hence a good model fit. According to the plotting, the PRM extremely underpredicted at the count of zero and overpredicted at both counts one and two. Therefore, we first eliminated the PRM. The other three models appeared to perform similarly and relatively well for the count of zero and count greater than two. At the count of one, the ZIP appeared to perform better than the NBRM and ZINB. In addition, compared with other models (see Table 2 for detailed model fit indices), the ZIP model also demonstrated a lower Akaike information criterion (AIC; 636.89), the smallest maximum difference from the observed (0.023), and the smallest mean difference between the residuals and the observed (0.009). Therefore, we chose to use the ZIP model to estimate the count variable, the count of different sources of support sought for IPV. When using a ZIP model, results are expected to contain two distinct components: the count component and the zero-inflated component. The count component identifies factors associated with the increase in the total number of different sources of support sought. The zero-inflated component predicts what factors specifically contribute to the “zeros;” in this study, the zero-inflated model identified factors contributing to participants’ seeking “zero” help (i.e., had not sought help from any sources) regardless of whether they contemplated the idea of help-seeking.

**Table 2.** Fit Indices and Comparisons of Predicted and Observed Probabilities of Counts in the Sample.

Model	LL	AIC	Maximum difference	Mean difference
PRM	−392.62	835.24	0.209 at count 0	0.055
NBRM	−302.20	656.41	−0.047 at count 1	0.014
**ZIP**	−**268.45**	**636.89**	**0.023 at count 3**	**0.009**
ZINB	−268.29	638.58	0.028 at count 3	0.012

*Note.* PRM = Poisson regression model; NBRM = negative binomial regression model; ZIP = zero-inflated Poisson model; ZINB = zero-inflated negative binomial model; LL = log-likelihood; AIC = Akaike information criterion.

## Results

### Results of the Multinomial Regression Model

Table 3 presents the results of the multinomial regression analysis. The dependent variables included three different help-seeking decisions: (a) never thought about seeking help (*n* = 102, 36.4%), (b) thought about seeking help but did not take action (*n* = 100, 35.7%), and (c) sought help for IPV (*n* = 78, 27.9%). We used the subgroup of participants who reported never thought about seeking help for IPV as the reference group. Model 1 compared those who thought about seeking help but did not take action with the reference group. Specifically, migrant women who broke up with their partner due to IPV (aOR = 0.32; *p* = .001) were less likely to contemplate help-seeking. Participants who reported having experienced sexual violence (aOR = 3.57; *p* = .008) were more likely to contemplate the idea of seeking help. Model 2 compared migrant women who sought help for IPV with the reference group. Having completed graduate school (relative to below college; aOR = 0.17; *p* = .005) and full-time employment status (relative to non-full-time employment status; aOR = 0.42; *p* = .048) were associated with decreased odds of seeking help for IPV. Finally, migrant women who self-owned or rented an apartment (relative to sharing housing with others; aOR = 2.58, *p* = .025), those who were 30 years old or younger (relative to 31 years old or older; aOR = 2.5, *p* = .013), and those who reported having more knowledge of the current anti-DV Law (aOR = 3.43, *p* = .001) were more likely to seek help for IPV.

**Table 3.** Results of Multinomial Regression Analysis by Help-Seeking Decision (*N* = 280).

	Model 1			Model 2		
Variables	aOR	95% CI	*p*	aOR	95% CI	*p*
Age	0.97	[0.91, 1.02]	0.237	0.95	[0.9, 1.01]	0.118
Marital status (Married as ref.)						
In a dating relationship	0.53	[0.17, 1.66]	0.271	0.75	[0.21, 2.65]	0.654
Cohabiting	0.8	[0.24, 2.7]	0.719	0.82	[0.21, 3.3]	0.786
Recent relationship ended/divorced	1.04	[0.26, 4.12]	0.958	1.72	[0.38, 7.86]	0.484
Partner contact (Monthly/only on holidays as ref.)						
Daily	0.42	[0.16, 1.06]	0.066	0.6	[0.22, 1.67]	0.331
Weekly	0.81	[0.31, 2.09]	0.659	0.94	[0.33, 2.66]	0.905
Broke up after IPV (Yes)	0.32***	[0.16, 0.64]	0.001	0.74	[0.35, 1.57]	0.427
Number of children^a^ (1 or more as ref.)						
0	2.84	[0.97, 8.38]	0.058	2.84	[0.86, 9.36]	0.087
Educational level^a^ (Below college as ref.)						
Completed college	0.94	[0.41, 2.15]	0.876	1.28	[0.53, 3.07]	0.585
Completed graduate school or above	0.34	[0.11, 1.05]	0.062	0.17**	[0.05, 0.58]	0.005
Employment^a^ (Non-full-time as ref.)						
Full-time	0.93	[0.4, 2.14]	0.859	0.42*	[0.18, 0.99]	0.048
Income^a^ (Below 4,000 Chinese Yuan as ref.)						
4,001–8,000 Chinese Yuan	1.27	[0.56, 2.92]	0.567	1.93	[0.79, 4.75]	0.15
8,001 Chinese Yuan or above	0.82	[0.28, 2.4]	0.715	1.64	[0.52, 5.12]	0.395
Current living condition^a^ (Sharing or living with others/Other as ref.)						
Self-owned/self-rental	1.66	[0.77, 3.56]	0.195	2.58*	[1.12, 5.92]	0.025
Residency before migration (Urban as ref.)						
Rural	0.88	[0.43, 1.8]	0.725	1.01	[0.47, 2.17]	0.986
Age first migrated for work^a^ (31 or older as ref.)						
30 or younger	0.9	[0.45, 1.8]	0.766	2.5*	[1.21, 5.17]	0.013
Number of cities ever migrated to^a^ (3 or more as ref.)						
1 to 2	0.78	[0.42, 1.48]	0.452	1.44	[0.73, 2.87]	0.296
IPV victimization						
Psychological aggression (No as ref.)	1.98	[0.93, 4.23]	0.077	0.7	[0.31, 1.56]	0.38
Controlling (No as ref.)	0.66	[0.26, 1.69]	0.389	1.07	[0.39, 2.91]	0.899
Physical violence (No as ref.)	0.47	[0.15, 1.48]	0.199	0.27	[0.08, 0.97]	0.044
Sexual violence (No as ref.)	3.57**	[1.4, 9.13]	0.008	2.73	[0.94, 7.96]	0.065
Belief that IPV is a private matter	1.04	[0.49, 2.21]	0.912	1.94	[0.83, 4.54]	0.125
Belongingness to the current city (No as ref.)	0.84	[0.39, 1.8]	0.647	0.86	[0.4, 1.87]	0.706
Belief that policies are effective	1.81	[0.8, 4.1]	0.154	2	[0.87, 4.59]	0.104
Knowledge of the anti-DV Law						
Had some or comprehensive knowledge	1.75	[0.88, 3.47]	0.109	3.43***	[1.63, 7.21]	0.001
Never heard of it/Heard of it but don’t know much (ref.)						

*Note.* ref. = reference group; IPV = intimate partner violence; aOR = adjusted odds ratio; Model 1 = Thought about seeking help but did not take actions vs. Never thought about seeking help (ref.); Model 2 = Sought help for IPV vs. Never thought about seeking help (ref.).

a These variables were collapsed into fewer categories to ensure adequate cell sizes for more robust analyses: For number of Children, 1 or more = *1/2/3/4 or more*. For employment, non-full-time = *Part-time/unemployed/Other*. For educational level, below college = *Completed middle school/Completed high school/Obtained an associate diploma*. For income, below 4,000 Chinese Yuan = *2,000 Chinese Yuan or below/2,001–4,000 Chinese Yuan*; 4,001–8,000 Chinese Yuan = *4,001–6,000 Chinese Yuan/6,001–8,000 Chinese Yuan;* 8,001 Chinese Yuan or above = *8,001–10,000 Chinese Yuan/10,000 Chinese Yuan or above.* For age first migrated for work, 30 years or younger = *17 years or below/18–30 years*; 31 or older = *31–40/41–50/51 or above*.

For number of cities ever migrated to, 3 or more = *3 to 4/5 or more*.

* *p* < .05; **p < .01; ***p < .001.

### Results of the ZIP Model

Table 4 shows the results of the ZIP model. Two factors were found to be associated with increased odds of seeking a greater number of different support sources: experiencing sexual violence (aOR = 2.49, *p* = .005) and having the belief that current policies effectively protect my rights (aOR = 2.39, *p* = .000). Four variables were associated with decreased odds of using more different support sources. These four variables were (a) being in a cohabiting relationship (compared with being married; aOR = 0.44, *p* = .042), (b) having an income level between 4,001–8,000 Chinese Yuan (compared with below 4,000 Chinese Yuan; aOR = 0.54, *p* = .012), (c) having an income level above 8,001 Chinese Yuan (aOR = 0.37, *p* = .007), and (d) having migrated to 1 or 2 cities (compared with 3 or more cities; aOR = 0.61, *p* = .02). In the zero-inflated model, those migrant women with a full-time job showed increased odds of not seeking help for IPV (aOR = 3.14, *p* = .028). Those who reported having some or comprehensive knowledge of the current anti-DV Law showed decreased odds of not seeking support for IPV (aOR = .29, *p* = .006).

**Table 4.** Results of Zero-Inflated Regression Model (*N* = 280).

	Count model			Zero-inflated model		
Variables	aOR	95% CI	*p*		95% CI	*p*
Age	0.96	[0.92, 1]	0.069	0.99	[0.89, 1.1]	0.871
Marital status (Married as ref.)						
In a dating relationship	0.75	[0.38, 1.47]	0.400	0.70	[0.17, 2.9]	0.627
Cohabiting	0.44*	[0.2, 0.97]	0.042	0.76	[0.16, 3.69]	0.733
Recent relationship ended/divorced	0.75	[0.34, 1.62]	0.460	0.41	[0.07, 2.37]	0.317
Partner contact (Monthly/only on holidays as ref.)						
Daily	1.00	[0.56, 1.79]	0.998	1.07	[0.35, 3.31]	0.904
Weekly	0.72	[0.43, 1.2]	0.203	0.79	[0.24, 2.59]	0.693
Broke up after IPV (Yes)	0.90	[0.59, 1.39]	0.645	0.81	[0.32, 2.02]	0.648
Number of children^a^ (1 or more as ref.)						
0	1.73	[0.96, 3.13]	0.069	0.73	[0.18, 3.02]	0.660
Educational level^a^ (Below college as ref.)						
Completed college	0.84	[0.55, 1.29]	0.427	0.54	[0.17, 1.75]	0.305
Completed graduate school or above	0.60	[0.28, 1.3]	0.197	4.42	[0.9, 21.78]	0.067
Employment^a^ (non-full-time as ref.)						
Full-time	0.96	[0.63, 1.48]	0.859	3.14*	[1.13, 8.66]	0.028
Income^a^ (Below 4,000 as ref.)						
4,001–8,000 Chinese Yuan	0.54*	[0.33, 0.87]	0.012	0.33	[0.1, 1.13]	0.078
8,001 Chinese Yuan or above	0.37**	[0.18, 0.76]	0.007	0.16	[0.01, 1.83]	0.140
Current living condition (Sharing or living with others/Other as ref.)						
Self-owned/self-rental	0.99	[0.64, 1.52]	0.954	0.52	[0.2, 1.34]	0.177
Residency before migration (Urban as ref.)						
Rural	0.80	[0.47, 1.37]	0.422	0.53	[0.11, 2.56]	0.430
Age first migrated for work^a^ (31 or older as ref.)						
30 or younger	1.40	[0.92, 2.15]	0.120	0.52	[0.19, 1.44]	0.208
Number of cities ever migrated to^a^ (3 or more as ref.)						
1 to 2	0.61*	[0.4, 0.93]	0.020	0.36	[0.11, 1.22]	0.102
IPV victimization						
Psychological aggression (No as ref.)	1.14	[0.69, 1.87]	0.610	3.22	[0.93, 11.1]	0.064
Controlling (No as ref.)	0.52	[0.24, 1.13]	0.096	0.28	[0.03, 3.05]	0.298
Physical violence (No as ref.)	3.05	[0.97, 9.59]	0.057	6.61	[0.58, 74.74]	0.127
Sexual violence (No as ref.)	2.49**	[1.32, 4.72]	0.005	1.19	[0.4, 3.56]	0.756
Belief that IPV is a private matter (No as ref.)	1.16	[0.7, 1.92]	0.565	0.51	[0.19, 1.33]	0.166
Belongingness to the current city (No as ref.)	1.10	[0.7, 1.71]	0.682	1.10	[0.45, 2.72]	0.835
Belief that policies are effective	2.39***	[1.48, 3.88]	0.000	1.08	[0.38, 3.07]	0.881
Knowledge of the anti-DV Law						
Had some or comprehensive knowledge	0.95	[0.64, 1.42]	0.817	0.29**	[0.12, 0.7]	0.006
Never heard of it / Heard of it but don’t know much (ref.)						
Log likelihood	−266.93		—	—	—	—
χ^2^	61.08***		—	—	—	—

*Note*. ref. = used as the reference group; IPV = intimate partner violence.

a These variables were collapsed into fewer categories to ensure adequate cell sizes for more robust analyses: For number of Children, 1 or more = *1/2/3/4 or more*. For employment, non-full-time = *Part-time/unemployed/Other*. For educational level, below college = *Completed middle school/Completed high school/Obtained an associate diploma*. For income, below 4,000 Chinese Yuan = *2,000 Chinese Yuan or below/2,001–4,000 Chinese Yuan*; 4,001–8,000 Chinese Yuan = *4,001–6,000 Chinese Yuan/6,001–8,000 Chinese Yuan;* 8,001 Chinese Yuan or above = *8,001–10,000 Chinese Yuan/10,000 Chinese Yuan or above.* For age first migrated for work, 30 years or younger = *17 years or below/18–30 years*; 31 years or older = *31–40 years/41–50 years/51 years or above*.

For number of cities ever migrated to, 3 or more = *3 to 4/5 or more*.

* *p* < .05; **p < .01; ***p < .001.

## Discussion and Implications

This study is one of the first studies to examine the help-seeking decisions of IPV survivors and their use of different sources of support in China. Using a sample of migrant women who are IPV survivors and reside in urban regions of China, the study found a relatively low rate of women who sought help after experiencing IPV (27.9%). Although over one-third of the women (35.7%) reported that they contemplated the idea of seeking help, they did not take further action beyond having the thought of help-seeking, which suggests that the participants may have cognitively recognized a need for support, but their needs were unmet; they might have had other concerns that prevented them from seeking support. Factors that stood out as significant in influencing their help-seeking decisions included socioeconomic factors, IPV type, relationship-related factors, knowledge of the anti-DV Law, and perception of the effectiveness of current policies.

Our findings suggest that having knowledge of the National anti-DV Law greatly increases the chance of help-seeking for IPV in domestic migrant women. In addition, migrant women were found to be more likely to seek help from more diverse sources of support when they believed that the current policies were effective in protecting their rights. We collected the data in early 2018 when the anti-DV Law had been enacted for 2 years in Mainland China. However, findings reveal that the majority of the migrant women in the study either had very limited knowledge (51%) or never heard of the law (14%). These results imply that social interventions should focus on raising awareness of the existence of the laws and increasing the knowledge of the laws among migrant women to further facilitate their help-seeking decisions. In addition, given the significant role of women’s belief in the effectiveness of current policies, it is important to strengthen the connections between legal professionals (e.g., lawyers, judges, and court officials) and IPV survivors and continue to build survivors’ trust in these sources of support and professionals, to promote help-seeking for IPV among migrant women.

Previous studies have reported the impacts of socioeconomic status on IPV disclosure or help-seeking. Some studies reported that higher socioeconomic status facilitated IPV disclosure and help-seeking, such as higher education and income levels (Ergocmen et al., 2013; Parvin et al., 2016; Tenkorang et al., 2016), and being employed (Goodson & Hayes, 2021; Linos et al., 2014; Vives-Cases & La Parra, 2017). Our findings, however, are somewhat inconsistent with these studies. Specifically, we found that a full-time job status and having completed graduate school were two significant factors that decreased migrant women’s odds of seeking help for IPV. Although feeling shameful or having a fear of “losing face” could be one possible explanation for not seeking help in the Chinese context (Xue, Fang, et al., 2019), a recent study examining reasons for not seeking help for IPV among Chinese women found that survivors with higher education were less likely to attribute their non-help-seeking to the belief that IPV is a “domestic shame” (Hu et al., 2021), which implies that there may have been other reasons or concerns associated with their non-help-seeking decision. One U.S.-based study examined women’s reasons for not seeking help and reported that compared with unemployed women, those employed were more likely to have other concerns, rather than “not needing help,” when it comes to reporting IPV to the police (Leone et al., 2014). Overall, future studies should continue to examine specific reasons for non-help-seeking among women with higher socioeconomic status.

In addition, we found that income was not significant in predicting women’s help-seeking. However, among those women who sought help, those with higher levels of income sought help from fewer number of different types of support. Previous studies have documented that women with higher income tend to seek specific forms of formal support, such as using legal services, seeking protection orders, and reporting IPV incidents to the police (Cattaneo et al., 2008; Durfee & Messing, 2012; Vatnar & Bjorkly, 2009). Furthermore, women with better income might also possess more social and economic resources to be more effective in locating appropriate sources of support to solve their concerns and challenges caused by IPV, and hence, a widespread outreach for many forms of help may not be necessary. For instance, those with higher income may be able to directly find and afford a private attorney to provide in-court legal representation or to file an order of protection. However, women with less financial freedom may need to rely on multiple sources of support, such as family, friends, and social service organizations, to be able to pursue legal justice (Durfee & Messing, 2012). Future studies should continue to understand how socioeconomic status may play a role in women’s help-seeking decisions and use of different types of support.

We found that migrant women who broke up with their partners due to IPV were more likely to seek help. In other words, this finding suggests that leaving an abusive relationship may decrease survivors’ concerns that could have prevented them from seeking help. This finding is somewhat consistent with a previous study on Chinese women’s help-seeking for IPV: ending an abusive intimate relationship was associated with an increased likelihood of seeking help (Hu et al., 2021). Studies also found that married women, compared with those who are not in a marriage, were less likely to seek help (Linos et al., 2014). It is possible that when IPV survivors continue to choose to stay in an abusive intimate relationship, their interpretation of the nature of IPV may always be changing, such as shifting from recognizing the emotional and physical hurts brought about by their abusive partners to attempting to validate or justify certain violent acts perpetrated by their intimate partners, a cognitive process which can make help-seeking a difficult decision (Liang et al., 2005). Alternatively, when survivors are committed to leaving an abusive relationship, they may have already passed the stage of contemplation and are more ready to take action when they recognize a need for external support.

Finally, that those migrant women victimized by sexual violence were more likely to contemplate the idea of seeking help implies a recognition of the seriousness of sexual IPV. Some previous studies have found that survivors of sexual IPV were more hesitant to seek help due to stigma, shame, or the normalization of sexual violence between intimate partners (e.g., McCleary-Sills et al., 2016). In the present sample, however, those having experienced sexual violence sought help from a broader range of sources of support. A recent review synthesized the impact of sexual IPV and revealed that “exposure to sexual violence” more than other forms of IPV elevates the risk of a variety of health and mental health issues, such as PTSD, physical pain, and reproductive health concerns (Barker et al., 2019). Therefore, it is likely that survivors of sexual IPV need to address a greater variety of needs. This situation may have contributed to the growing number of diverse forms of support to which these survivors reached out. The present study did not assess help-seeking outcomes; therefore, it remains unknown how much of their decision to reach out for more sources of help was, in fact, a result of not being able to locate the most suitable type of support and hence the need to reach out to multiple different sources of support. In recent years, the ongoing feminist movement in urban areas in China has focused on addressing sexual violence against women by challenging prevailing sexual norms and sexist practices (e.g., Zheng, 2015). In addition, an increasing number of feminist advocacy groups have moved their activist campaigns onto Chinese mainstream social media, greatly expanding the scope of their influence to other non-urban regions of China (Wang & Driscoll, 2019). This continued anti-sexual harassment advocacy, working in tandem with the passage of the Anti-DV Law, might have promoted women’s motivation to seek help after experiencing sexual violence. These possible explanations, however, need to be further researched in the context of contemporary China. Future studies may consider further exploring the needs of sexual IPV survivors, asking such questions as (a) how their needs drive their coping strategies and seeking different forms of support, (b) how the effectiveness of help-seeking may influence their future help-seeking decisions, and (c) how the larger sociopolitical context (e.g., the passage of the anti-DV Law and the rising anti-sexual harassment movement and social media campaigns) may play a role in their help-seeking for IPV.

## Limitations

Several limitations exist in the present study. First, given the nonprobability sampling procedure, our findings are not representative of all migrant women in urban cities in China. Specifically, the majority of the women in our sample were employed (i.e., 72 and 14% were full-time and part-time workers, respectively). Over half of the sample had completed college or graduate school. Since the majority of Chinese migrants in urban areas merely complete high school (UNFPA, 2019), socioeconomically disadvantaged migrant women are likely underrepresented in the present sample. In addition, the use of a questionnaire hosted online might have only solicited participation from those with internet access. Survivors from rural regions and those experiencing partner control might not have been able to participate in the study. Second, the severity of IPV has been identified as a significant factor associated with IPV help-seeking in some studies (e.g., Choi et al., 2018; Goodson & Hayes, 2018; Rowan et al., 2021). In the current study, however, we did not measure the severity and frequency of migrant women’s victimization. Finally, the cross-sectional nature of the study design does not allow us to identify causal relationships, such as inferring further whether some of the significant factors (e.g., more knowledge of laws) facilitate help-seeking or are a result of having received some sources of support, such as friends or lawyers.

## Conclusion

The present study is one of the first to examine the help-seeking decisions among Chinese migrant women who experience IPV. We found a low rate of help-seeking and a moderate rate of having contemplated the idea of seeking help. This study offers insights for promoting migrant women’s help-seeking after experiencing IPV, such as (a) raising awareness of, and building their trust in, the existing policies and laws, (b) providing more targeted resources for this population, especially for those socioeconomically disadvantaged, and (c) providing sufficient support for IPV-affected women who are in a marriage or in a cohabiting relationship, especially when they are not ready to leave an abusive relationship.
